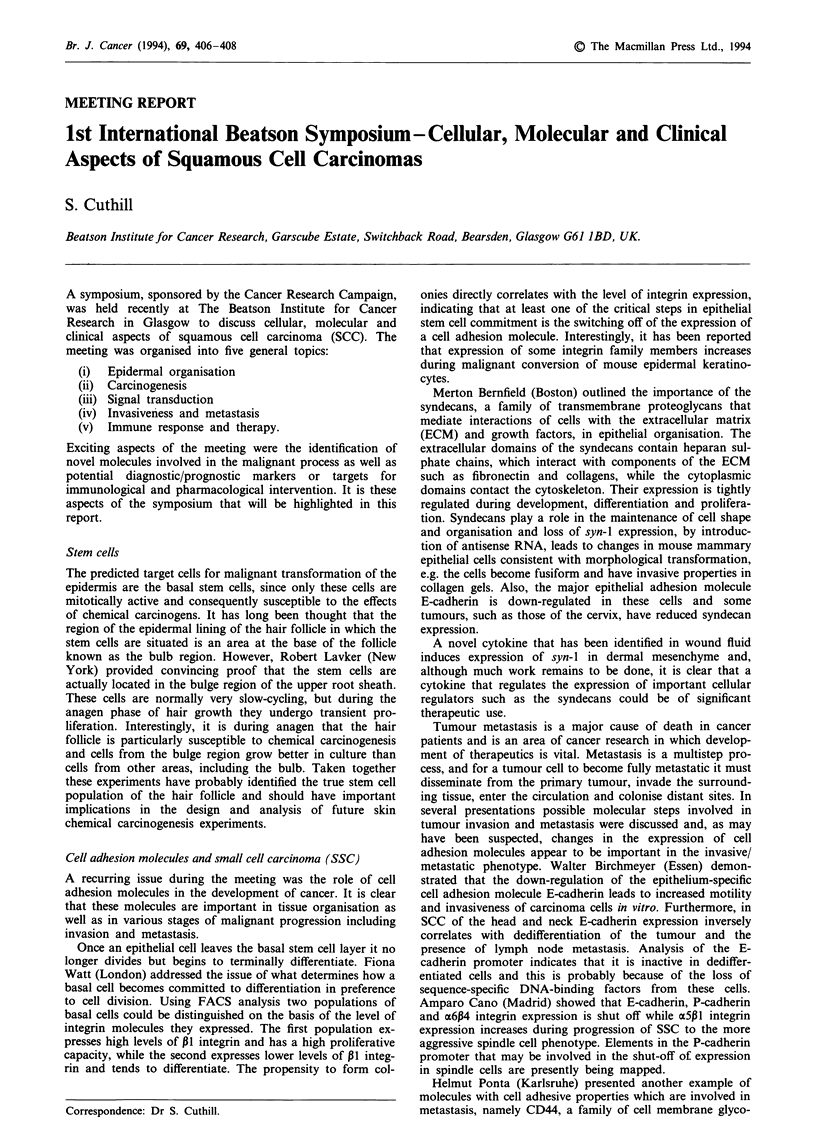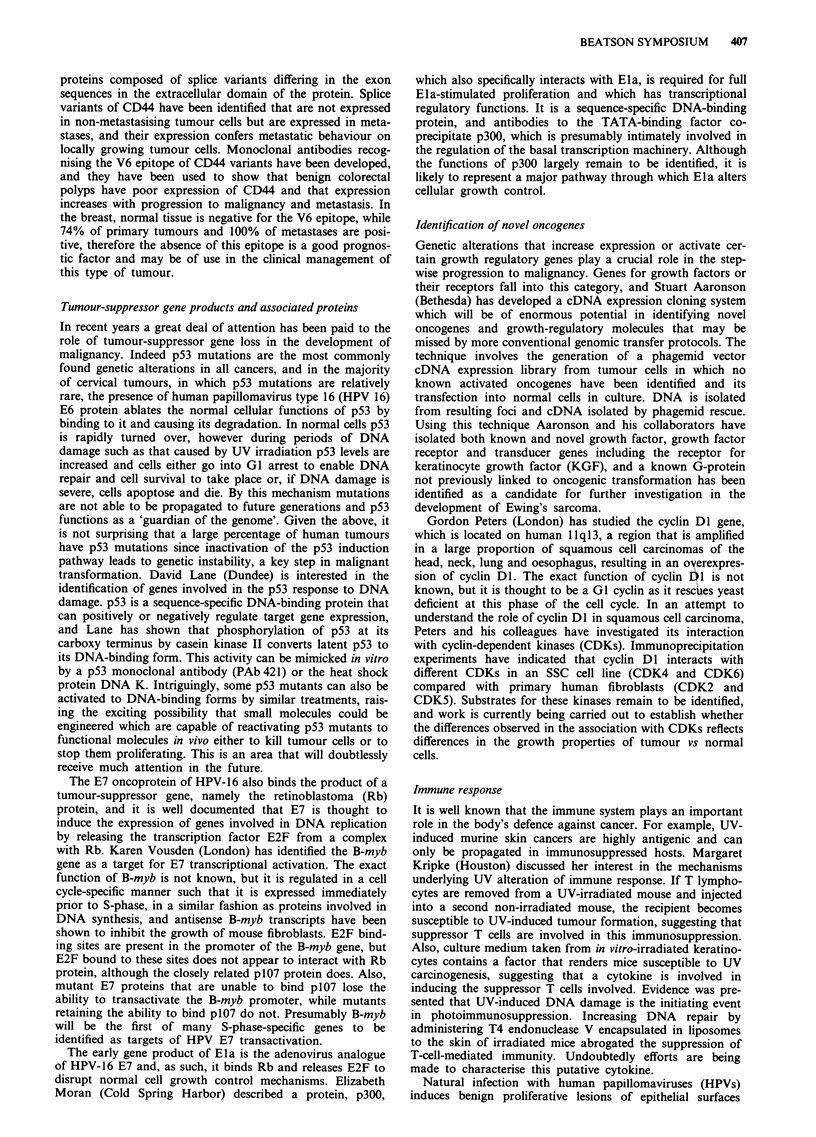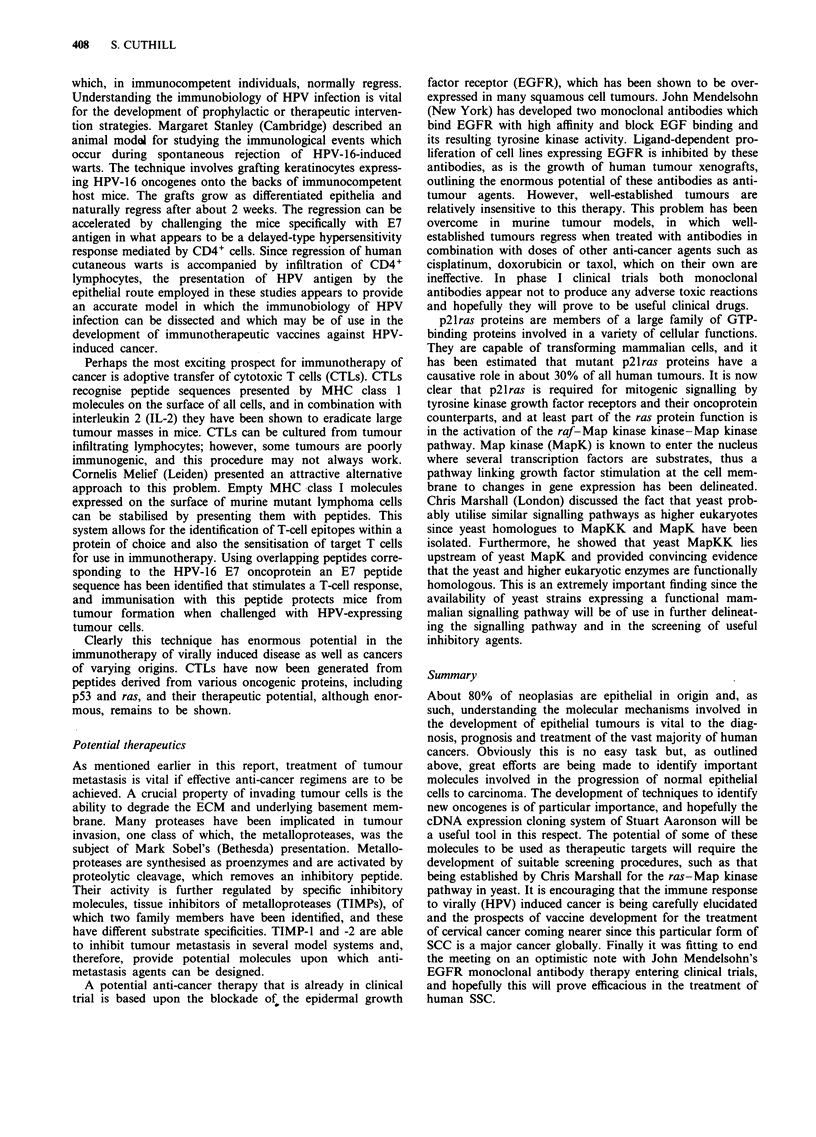# 1st international Beatson symposium--cellular, molecular and clinical aspects of squamous cell carcinomas.

**DOI:** 10.1038/bjc.1994.75

**Published:** 1994-02

**Authors:** S. Cuthill

**Affiliations:** Beatson Institute for Cancer Research, Bearsden, Glasgow, UK.

## Abstract

About 80% of neoplasias are epithelial in origin and, as such, understanding the molecular mechanisms involved in the development of epithelial tumours is vital to the diagnosis, prognosis and treatment of the vast majority of human cancers. Obviously this is no easy task but, as outlined above, great efforts are being made to identify important molecules involved in the progression of normal epithelial cells to carcinoma. The development of techniques to identify new oncogenes is of particular importance, and hopefully the cDNA expression cloning system of Stuart Aaronson will be a useful tool in this respect. The potential of some of these molecules to be used as therapeutic targets will require the development of suitable screening procedures, such as that being established by Chris Marshall for the ras-Map kinase pathway in yeast. It is encouraging that the immune response to virally (HPV) induced cancer is being carefully elucidated and the prospects of vaccine development for the treatment of cervical cancer coming nearer since this particular form of SCC is a major cancer globally. Finally it was fitting to end the meeting on an optimistic note with John Mendelsohn's EGFR monoclonal antibody therapy entering clinical trials, and hopefully this will prove efficacious in the treatment of human SSC.


*Br. J. Cancer* (1994), **69**, 406–408 © The Macmillan Press Ltd., 1994

MEETING REPORT

### 1st International Beatson Symposium – Cellular, Molecular and Clinical Aspects of Squamous Cell Carcinomas

S. Cuthill

*Beatson Institute for Cancer Research, Garscube Estate, Switchback Road, Bearsden, Glasgow G61 1BD, UK.*

A symposium, sponsored by the Cancer Research Campaign, was held recently at The Beatson Institute for Cancer Research in Glasgow to discuss cellular, molecular and clinical aspects of squamous cell carcinoma (SCC). The meeting was organised into five general topics:

(i) Epidermal organisation
(ii) Carcinogenesis
(iii) Signal transduction
(iv) Invasiveness and metastasis
(v) Immune response and therapy.

Exciting aspects of the meeting were the identification of novel molecules involved in the malignant process as well as potential diagnostic/prognostic markers or targets for immunological and pharmacological intervention. It is these aspects of the symposium that will be highlighted in this report.

### *Stem cells*

The predicted target cells for malignant transformation of the epidermis are the basal stem cells, since only these cells are mitotically active and consequently susceptible to the effects of chemical carcinogens. It has long been thought that the region of the epidermal lining of the hair follicle in which the stem cells are situated is an area at the base of the follicle known as the bulb region. However, Robert Lavker (New York) provided convincing proof that the stem cells are actually located in the bulge region of the upper root sheath. These cells are normally very slow-cycling, but during the anagen phase of hair growth they undergo transient proliferation. Interestingly, it is during anagen that the hair follicle is particularly susceptible to chemical carcinogenesis and cells from the bulge region grow better in culture than cells from other areas, including the bulb. Taken together these experiments have probably identified the true stem cell population of the hair follicle and should have important implications in the design and analysis of future skin chemical carcinogenesis experiments.

### *Cell adhesion molecules and small cell carcinoma (SSC)*

A recurring issue during the meeting was the role of cell adhesion molecules in the development of cancer. It is clear that these molecules are important in tissue organisation as well as in various stages of malignant progression including invasion and metastasis.

Once an epithelial cell leaves the basal stem cell layer it no longer divides but begins to terminally differentiate. Fiona Watt (London) addressed the issue of what determines how a basal cell becomes committed to differentiation in preference to cell division. Using FACS analysis two populations of basal cells could be distinguished on the basis of the level of integrin molecules they expressed. The first population expresses high levels of $\beta 1$ integrin and has a high proliferative capacity, while the second expresses lower levels of $\beta 1$ integrin and tends to differentiate. The propensity to form colonies directly correlates with the level of integrin expression, indicating that at least one of the critical steps in epithelial stem cell commitment is the switching off of the expression of a cell adhesion molecule. Interestingly, it has been reported that expression of some integrin family members increases during malignant conversion of mouse epidermal keratinocytes.

Merton Bernfield (Boston) outlined the importance of the syndecans, a family of transmembrane proteoglycans that mediate interactions of cells with the extracellular matrix (ECM) and growth factors, in epithelial organisation. The extracellular domains of the syndecans contain heparan sulphate chains, which interact with components of the ECM such as fibronectin and collagens, while the cytoplasmic domains contact the cytoskeleton. Their expression is tightly regulated during development, differentiation and proliferation. Syndecans play a role in the maintenance of cell shape and organisation and loss of *syn*-1 expression, by introduction of antisense RNA, leads to changes in mouse mammary epithelial cells consistent with morphological transformation, e.g. the cells become fusiform and have invasive properties in collagen gels. Also, the major epithelial adhesion molecule E-cadherin is down-regulated in these cells and some tumours, such as those of the cervix, have reduced syndecan expression.

A novel cytokine that has been identified in wound fluid induces expression of *syn*-1 in dermal mesenchyme and, although much work remains to be done, it is clear that a cytokine that regulates the expression of important cellular regulators such as the syndecans could be of significant therapeutic use.

Tumour metastasis is a major cause of death in cancer patients and is an area of cancer research in which development of therapeutics is vital. Metastasis is a multistep process, and for a tumour cell to become fully metastatic it must disseminate from the primary tumour, invade the surrounding tissue, enter the circulation and colonise distant sites. In several presentations possible molecular steps involved in tumour invasion and metastasis were discussed and, as may have been suspected, changes in the expression of cell adhesion molecules appear to be important in the invasive/metastatic phenotype. Walter Birchmeyer (Essen) demonstrated that the down-regulation of the epithelium-specific cell adhesion molecule E-cadherin leads to increased motility and invasiveness of carcinoma cells *in vitro*. Furthermore, in SCC of the head and neck E-cadherin expression inversely correlates with dedifferentiation of the tumour and the presence of lymph node metastasis. Analysis of the E-cadherin promoter indicates that it is inactive in dedifferentiated cells and this is probably because of the loss of sequence-specific DNA-binding factors from these cells. Amparo Cano (Madrid) showed that E-cadherin, P-cadherin and $\alpha 6\beta 4$ integrin expression is shut off while $\alpha 5\beta 1$ integrin expression increases during progression of SSC to the more aggressive spindle cell phenotype. Elements in the P-cadherin promoter that may be involved in the shut-off of expression in spindle cells are presently being mapped.

Helmut Ponta (Karlsruhe) presented another example of molecules with cell adhesive properties which are involved in metastasis, namely CD44, a family of cell membrane glyco-

Correspondence: Dr S. Cuthill.

proteins composed of splice variants differing in the exon sequences in the extracellular domain of the protein. Splice variants of CD44 have been identified that are not expressed in non-metastasising tumour cells but are expressed in metastases, and their expression confers metastatic behaviour on locally growing tumour cells. Monoclonal antibodies recognising the V6 epitope of CD44 variants have been developed, and they have been used to show that benign colorectal polyps have poor expression of CD44 and that expression increases with progression to malignancy and metastasis. In the breast, normal tissue is negative for the V6 epitope, while 74% of primary tumours and 100% of metastases are positive, therefore the absence of this epitope is a good prognostic factor and may be of use in the clinical management of this type of tumour.

*Tumour-suppressor gene products and associated proteins*

In recent years a great deal of attention has been paid to the role of tumour-suppressor gene loss in the development of malignancy. Indeed p53 mutations are the most commonly found genetic alterations in all cancers, and in the majority of cervical tumours, in which p53 mutations are relatively rare, the presence of human papillomavirus type 16 (HPV 16) E6 protein ablates the normal cellular functions of p53 by binding to it and causing its degradation. In normal cells p53 is rapidly turned over, however during periods of DNA damage such as that caused by UV irradiation p53 levels are increased and cells either go into G1 arrest to enable DNA repair and cell survival to take place or, if DNA damage is severe, cells apoptose and die. By this mechanism mutations are not able to be propagated to future generations and p53 functions as a 'guardian of the genome'. Given the above, it is not surprising that a large percentage of human tumours have p53 mutations since inactivation of the p53 induction pathway leads to genetic instability, a key step in malignant transformation. David Lane (Dundee) is interested in the identification of genes involved in the p53 response to DNA damage. p53 is a sequence-specific DNA-binding protein that can positively or negatively regulate target gene expression, and Lane has shown that phosphorylation of p53 at its carboxy terminus by casein kinase II converts latent p53 to its DNA-binding form. This activity can be mimicked *in vitro* by a p53 monoclonal antibody (PAb 421) or the heat shock protein DNA K. Intriguingly, some p53 mutants can also be activated to DNA-binding forms by similar treatments, raising the exciting possibility that small molecules could be engineered which are capable of reactivating p53 mutants to functional molecules *in vivo* either to kill tumour cells or to stop them proliferating. This is an area that will doubtlessly receive much attention in the future.

The E7 oncoprotein of HPV-16 also binds the product of a tumour-suppressor gene, namely the retinoblastoma (Rb) protein, and it is well documented that E7 is thought to induce the expression of genes involved in DNA replication by releasing the transcription factor E2F from a complex with Rb. Karen Vousden (London) has identified the B-*myb* gene as a target for E7 transcriptional activation. The exact function of B-*myb* is not known, but it is regulated in a cell cycle-specific manner such that it is expressed immediately prior to S-phase, in a similar fashion as proteins involved in DNA synthesis, and antisense B-*myb* transcripts have been shown to inhibit the growth of mouse fibroblasts. E2F binding sites are present in the promoter of the B-*myb* gene, but E2F bound to these sites does not appear to interact with Rb protein, although the closely related p107 protein does. Also, mutant E7 proteins that are unable to bind p107 lose the ability to transactivate the B-*myb* promoter, while mutants retaining the ability to bind p107 do not. Presumably B-*myb* will be the first of many S-phase-specific genes to be identified as targets of HPV E7 transactivation.

The early gene product of E1a is the adenovirus analogue of HPV-16 E7 and, as such, it binds Rb and releases E2F to disrupt normal cell growth control mechanisms. Elizabeth Moran (Cold Spring Harbor) described a protein, p300, which also specifically interacts with E1a, is required for full E1a-stimulated proliferation and which has transcriptional regulatory functions. It is a sequence-specific DNA-binding protein, and antibodies to the TATA-binding factor coprecipitate p300, which is presumably intimately involved in the regulation of the basal transcription machinery. Although the functions of p300 largely remain to be identified, it is likely to represent a major pathway through which E1a alters cellular growth control.

*Identification of novel oncogenes*

Genetic alterations that increase expression or activate certain growth regulatory genes play a crucial role in the stepwise progression to malignancy. Genes for growth factors or their receptors fall into this category, and Stuart Aaronson (Bethesda) has developed a cDNA expression cloning system which will be of enormous potential in identifying novel oncogenes and growth-regulatory molecules that may be missed by more conventional genomic transfer protocols. The technique involves the generation of a phagemid vector cDNA expression library from tumour cells in which no known activated oncogenes have been identified and its transfection into normal cells in culture. DNA is isolated from resulting foci and cDNA isolated by phagemid rescue. Using this technique Aaronson and his collaborators have isolated both known and novel growth factor, growth factor receptor and transducer genes including the receptor for keratinocyte growth factor (KGF), and a known G-protein not previously linked to oncogenic transformation has been identified as a candidate for further investigation in the development of Ewing's sarcoma.

Gordon Peters (London) has studied the cyclin D1 gene, which is located on human 11q13, a region that is amplified in a large proportion of squamous cell carcinomas of the head, neck, lung and oesophagus, resulting in an overexpression of cyclin D1. The exact function of cyclin D1 is not known, but it is thought to be a G1 cyclin as it rescues yeast deficient at this phase of the cell cycle. In an attempt to understand the role of cyclin D1 in squamous cell carcinoma, Peters and his colleagues have investigated its interaction with cyclin-dependent kinases (CDKs). Immunoprecipitation experiments have indicated that cyclin D1 interacts with different CDKs in an SSC cell line (CDK4 and CDK6) compared with primary human fibroblasts (CDK2 and CDK5). Substrates for these kinases remain to be identified, and work is currently being carried out to establish whether the differences observed in the association with CDKs reflects differences in the growth properties of tumour *vs* normal cells.

*Immune response*

It is well known that the immune system plays an important role in the body's defence against cancer. For example, UV-induced murine skin cancers are highly antigenic and can only be propagated in immunosuppressed hosts. Margaret Kripke (Houston) discussed her interest in the mechanisms underlying UV alteration of immune response. If T lymphocytes are removed from a UV-irradiated mouse and injected into a second non-irradiated mouse, the recipient becomes susceptible to UV-induced tumour formation, suggesting that suppressor T cells are involved in this immunosuppression. Also, culture medium taken from *in vitro*-irradiated keratinocytes contains a factor that renders mice susceptible to UV carcinogenesis, suggesting that a cytokine is involved in inducing the suppressor T cells involved. Evidence was presented that UV-induced DNA damage is the initiating event in photoimmunosuppression. Increasing DNA repair by administering T4 endonuclease V encapsulated in liposomes to the skin of irradiated mice abrogated the suppression of T-cell-mediated immunity. Undoubtedly efforts are being made to characterise this putative cytokine.

Natural infection with human papillomaviruses (HPVs) induces benign proliferative lesions of epithelial surfaces which, in immunocompetent individuals, normally regress. Understanding the immunobiology of HPV infection is vital for the development of prophylactic or therapeutic intervention strategies. Margaret Stanley (Cambridge) described an animal model for studying the immunological events which occur during spontaneous rejection of HPV-16-induced warts. The technique involves grafting keratinocytes expressing HPV-16 oncogenes onto the backs of immunocompetent host mice. The grafts grow as differentiated epithelia and naturally regress after about 2 weeks. The regression can be accelerated by challenging the mice specifically with E7 antigen in what appears to be a delayed-type hypersensitivity response mediated by $CD4^+$ cells. Since regression of human cutaneous warts is accompanied by infiltration of $CD4^+$ lymphocytes, the presentation of HPV antigen by the epithelial route employed in these studies appears to provide an accurate model in which the immunobiology of HPV infection can be dissected and which may be of use in the development of immunotherapeutic vaccines against HPV-induced cancer.

Perhaps the most exciting prospect for immunotherapy of cancer is adoptive transfer of cytotoxic T cells (CTLs). CTLs recognise peptide sequences presented by MHC class 1 molecules on the surface of all cells, and in combination with interleukin 2 (IL-2) they have been shown to eradicate large tumour masses in mice. CTLs can be cultured from tumour infiltrating lymphocytes; however, some tumours are poorly immunogenic, and this procedure may not always work. Cornelis Melief (Leiden) presented an attractive alternative approach to this problem. Empty MHC class I molecules expressed on the surface of murine mutant lymphoma cells can be stabilised by presenting them with peptides. This system allows for the identification of T-cell epitopes within a protein of choice and also the sensitisation of target T cells for use in immunotherapy. Using overlapping peptides corresponding to the HPV-16 E7 oncoprotein an E7 peptide sequence has been identified that stimulates a T-cell response, and immunisation with this peptide protects mice from tumour formation when challenged with HPV-expressing tumour cells.

Clearly this technique has enormous potential in the immunotherapy of virally induced disease as well as cancers of varying origins. CTLs have now been generated from peptides derived from various oncogenic proteins, including p53 and *ras*, and their therapeutic potential, although enormous, remains to be shown.

### *Potential therapeutics*

As mentioned earlier in this report, treatment of tumour metastasis is vital if effective anti-cancer regimens are to be achieved. A crucial property of invading tumour cells is the ability to degrade the ECM and underlying basement membrane. Many proteases have been implicated in tumour invasion, one class of which, the metalloproteases, was the subject of Mark Sobel's (Bethesda) presentation. Metalloproteases are synthesised as proenzymes and are activated by proteolytic cleavage, which removes an inhibitory peptide. Their activity is further regulated by specific inhibitory molecules, tissue inhibitors of metalloproteases (TIMPs), of which two family members have been identified, and these have different substrate specificities. TIMP-1 and -2 are able to inhibit tumour metastasis in several model systems and, therefore, provide potential molecules upon which anti-metastasis agents can be designed.

A potential anti-cancer therapy that is already in clinical trial is based upon the blockade of the epidermal growth factor receptor (EGFR), which has been shown to be over-expressed in many squamous cell tumours. John Mendelsohn (New York) has developed two monoclonal antibodies which bind EGFR with high affinity and block EGF binding and its resulting tyrosine kinase activity. Ligand-dependent proliferation of cell lines expressing EGFR is inhibited by these antibodies, as is the growth of human tumour xenografts, outlining the enormous potential of these antibodies as anti-tumour agents. However, well-established tumours are relatively insensitive to this therapy. This problem has been overcome in murine tumour models, in which well-established tumours regress when treated with antibodies in combination with doses of other anti-cancer agents such as cisplatinum, doxorubicin or taxol, which on their own are ineffective. In phase I clinical trials both monoclonal antibodies appear not to produce any adverse toxic reactions and hopefully they will prove to be useful clinical drugs.

p21*ras* proteins are members of a large family of GTP-binding proteins involved in a variety of cellular functions. They are capable of transforming mammalian cells, and it has been estimated that mutant p21*ras* proteins have a causative role in about 30% of all human tumours. It is now clear that p21*ras* is required for mitogenic signalling by tyrosine kinase growth factor receptors and their oncoprotein counterparts, and at least part of the *ras* protein function is in the activation of the *raf*–Map kinase kinase–Map kinase pathway. Map kinase (MapK) is known to enter the nucleus where several transcription factors are substrates, thus a pathway linking growth factor stimulation at the cell membrane to changes in gene expression has been delineated. Chris Marshall (London) discussed the fact that yeast probably utilise similar signalling pathways as higher eukaryotes since yeast homologues to MapKK and MapK have been isolated. Furthermore, he showed that yeast MapKK lies upstream of yeast MapK and provided convincing evidence that the yeast and higher eukaryotic enzymes are functionally homologous. This is an extremely important finding since the availability of yeast strains expressing a functional mammalian signalling pathway will be of use in further delineating the signalling pathway and in the screening of useful inhibitory agents.

### *Summary*

About 80% of neoplasias are epithelial in origin and, as such, understanding the molecular mechanisms involved in the development of epithelial tumours is vital to the diagnosis, prognosis and treatment of the vast majority of human cancers. Obviously this is no easy task but, as outlined above, great efforts are being made to identify important molecules involved in the progression of normal epithelial cells to carcinoma. The development of techniques to identify new oncogenes is of particular importance, and hopefully the cDNA expression cloning system of Stuart Aaronson will be a useful tool in this respect. The potential of some of these molecules to be used as therapeutic targets will require the development of suitable screening procedures, such as that being established by Chris Marshall for the *ras*–Map kinase pathway in yeast. It is encouraging that the immune response to virally (HPV) induced cancer is being carefully elucidated and the prospects of vaccine development for the treatment of cervical cancer coming nearer since this particular form of SCC is a major cancer globally. Finally it was fitting to end the meeting on an optimistic note with John Mendelsohn's EGFR monoclonal antibody therapy entering clinical trials, and hopefully this will prove efficacious in the treatment of human SSC.